# Whole exome sequencing identifies somatically mutated genes in bladder cancer: A pilot study from Bangladesh

**DOI:** 10.1016/j.bbrep.2026.102574

**Published:** 2026-04-03

**Authors:** Hasib Uddin Ahmed, Md Raiyan Hosen, Munshi Akid Mostofa, Md Akmal Hossain, Md Omar Faruk, Hossain Uddin Shekhar, AKM Mahbub Hasan, Md Ismail Hosen

**Affiliations:** a Clinical Biochemistry and Translational Medicine Laboratory (CBTML), Department of Biochemistry and Molecular Biology, University of Dhaka, Dhaka, 1000, Bangladesh; b National Institute of Kidney Diseases and Urology, Dhaka, 1000, Bangladesh

**Keywords:** Bladder cancer, Whole-exome sequencing, Somatic mutations, Genomic profiling, Mutational signatures, Bangladesh

## Abstract

Bladder cancer, ranking as the tenth most common cancer globally, lacks genomic characterization in Bangladeshi patients. This study is an exploratory analysis of the genomic landscape of bladder cancer in this population by conducting whole-exome sequencing (WES) on tumor (median coverage = 65X) and matched blood (median coverage = 68X) samples from four patients at the National Institute of Cancer Research and Hospital in Dhaka, using the Illumina HiSeq platform. Somatic mutations were identified using GATK Mutect2 and analyzed with bioinformatics tools to explore potential genetic alterations in those four individuals. Single Nucleotide Variants (SNVs) were observed in *TP53* (25%, 1/4), *KMT2C* (75%, 3/4), and *FLG* (100%, 4/4), with mutations in *AHNAK2* (75%, 3/4) and *KCNJ18* (50%, 2/4) in this limited cohort. Pathway analysis highlighted possible disruptions in *RTK-RAS* and *NOTCH* signaling pathways, which are critical for cell proliferation and differentiation. Mutational signature analysis from the four sample data revealed the single base substitution (SBS2) signature, which is linked to *APOBEC* activity and SBS3 linked to defects in homologous recombination based DNA repair. Additionally, copy number variations (CNVs) were evaluated, identifying chromosomal losses (e.g., Y chromosome, 1p12, 9p21.2-p21.3) and gains (e.g., 1q21.3, 6p22.3). This study establishes a foundational dataset for Bangladeshi bladder cancer patients, paving the way for further validation experiments in vitro, and the possibility of developing population-specific molecular biomarker panels, these insights may also contribute to personalized treatment strategies and help combat rising morbidity and recurrence rates of bladder cancer in Bangladesh.

## Highlights

• First WES study in Bangladeshi bladder cancer patients.
• Detected mutations in TP53, KMT2C, and FLG within the cohort.
• Frequently mutated genes for the population: *AHNAK2* and *KCNJ18* mutations.
• Pathway disruptions in *RTK-RAS* and *NOTCH* signaling.
• *APOBEC*-related mutational signature detected.

## 1 Introduction

Bladder cancer is the 9th leading cause of cancer death in men, and the 10th most commonly diagnosed cancer in the world with about 614,000 new cases and 220,000 deaths in 2022 alone [1]. Globally, this particular type of cancer occurs approximately four times more in men than in women, in terms of both incidence and mortality rate [1]. Bladder cancer is associated with a high rate of morbidity, death, and financial costs [2]. Bladder cancer was also found to be one of the most frequently mutated human cancers, following lung and skin cancer in terms of mutation rates per Megabases of the human genome [3]. In Bangladesh, 961 bladder cancer associated deaths were reported in a 2020 WHO study [4]. 366 more patients died within the next two years, as evident in a 2022 WHO study, with 2.6 deaths per 100,000. The main risk factors for bladder cancer include exposure to carcinogens in the environment or at work, particularly tobacco and other chemical compounds [5].

The European Association of Urology (EAU) classifies bladder cancer into two categories: non-muscle invasive bladder cancer (NMIBC) which is defined as tumor stage T1, Ta or Tis and takes up about 70% of all cases, and muscular invasive bladder cancer (MIBC) or T2 tumor stage [6]. Worldwide, the incidence of stage Ta, T1 and Tis of NMIBC is 70%, 20% and 10% respectively [7] and this is the TNM (Tumor, Node and Metastasis) staging of NMIBC. Non-muscular invasive bladder cancer (NMIBC) patients generally have favorable cancer-specific survival and overall survival [8] although >50% of these patients face disease recurrence and approximately 20% suffer from disease progression to muscle invasive bladder cancer (MIBC) [9].

Europe, Middle East, and North Africa have higher occurrence and death rates for bladder cancer than South Asia. Nevertheless, incidence and death rates are on the rise in East and Central Asia [10]. This places South Asia as a particularly understudied population not only in terms of occurrence studies, but also in research and population genetics.

Bladder cancer damages the urothelial cells that line the urinary bladder's lumen. Tumors of the bladder, upper urinary tract and proximal urethra are all classified as urothelial carcinoma [11]. Tumor staging in this particular type of cancer is based on the extent of tumor invasion of the bladder wall [12]. Potential bladder cancer patients primarily suffer from microscopic or macroscopic hematuria. Other symptoms that may coexist with hematuria are abdominal pain, urinary frequency and Dysuria or painful urination.

Recurrent somatic mutations and chromosomal losses or gains could be targeted as potential biomarkers for noninvasive and early bladder cancer detection. TP53 mutations were revealed to be indicators of disease progression in patients with NMIBC and MIBC in early studies [13]. Common mutations in bladder cancer also include *PIK3CA, TSC1,*and *HRAS*. Mutations in *CDKN1A, CDKN1B* and *RBL1, KDM6A, FGFR3* are specifically indicative of NMIBC [14] [15] [16] while mutations in *P16, RB1, ERBB2, CDKN2A* and *KMT2A* genes are found in patients with MIBC [17] [18]. *TERT* promoter mutations are more frequent than any earlier reported gene in Bladder cancer [19]. Along with common mutations which are detected across multiple bladder cancer classifications, mutated genes that are specific to one kind of bladder cancer are pivotal in establishing early detection and personalized treatment. Multiple germline mutation studies have been conducted in Bangladeshi populations, where genes such as *TP53*, *NAT2* and *ERCC2* were commonly mutated in bladder cancer patients [20]. However, no somatic mutation studies from tissue samples have been conducted or reported yet.

Whole exome sequencing (WES) analyzes the 1-2% of the genome that contains the protein-coding regions, which harbors up to 85% of all known disease-causing mutations [21]. Sequencing depth and coverage are two parameters for sequencing quality measurement. More than 90-95% of the sequence should be covered at least 10-fold (10X) and the average sequence depth should be greater or equal to 100 folds (100X) [22]. This study was designed as a pilot investigation to assess the feasibility of whole-exome sequencing in Bangladeshi bladder cancer patients, given resource constraints, and limited availability of samples.

The primary objective of our study is to perform a pilot genomic characterization study of Bladder cancer in Bangladeshi patients. As an exploratory analysis, the specific objectives of the study include initiation of a database containing Bangladeshi bladder cancer patients’ information and biospecimen, identification of top somatic gene mutations and comparison with global cohorts, and analysis of mutational signatures and pathways. Identified mutations and genes should be subject to further validation through experiments due to the low sample count of this study. However, findings should provide future studies with reference to compare results. Additionally, findings on mutated genes, protein domains, hypermutation and mutational signatures would help direct research efforts. This is a novel study for Bangladesh, which may contribute to efforts to establish a biomarker panel establishment.

## 2 Materials and methods

### 2.1 Patient samples and DNA extraction

Four bladder cancer patients from the National Institute of Cancer Research and Hospital in Dhaka, Bangladesh, were enrolled with informed consent under an NICRH-approved protocol. Clinical and histopathological criteria were also collected. On the day of Transurethral Resection of Bladder Tumor (TURBT) surgery, 5 mL of peripheral blood was collected in EDTA tubes, and tumor tissues were preserved in RNA later (Qiagen). Tumor DNA was extracted using the Qiagen Puregene Core Kit A, while blood DNA was isolated via the organic phenol-chloroform method. DNA quality and concentration were assessed using agarose gel electrophoresis and NanoDrop. After purity inspection, DNA samples of the first four enrolled patients (Patient ID 001 to ID 004) were selected for Whole Exome Sequencing.

### 2.2 Whole exome sequencing (WES)

Paired-end WES was performed on tumor and matched normal (blood) DNA using the Illumina HiSeq platform. Exome enrichment was carried out with the Agilent SureSelect XT and V7-post capture kits, covering 99.7% of coding exons and achieving ≥30 million read-pairs per sample.

### 2.3 Bioinformatics workflow

To ensure our thorough analysis, each analytic and visualization step was selected to address a specific aspect of the study's aims, mainly detecting somatic variants, assessing their functional relevance, identifying perturbed pathways, evaluating mutational processes, and profiling copy number changes. These domains represent major genomic mechanisms known to drive bladder cancer development and progression.

#### 2.3.1 Quality control

FASTQ files were quality-checked using FASTQC (v0.11.9) and MultiQC to assess base quality, GC content, adapter contamination, and sequence duplication. Adapter trimming and quality filtering were performed with Trimmomatic (v0.39).

#### 2.3.2 Sequence alignment

Clean reads were aligned to the GRCh38/hg38 human reference genome using BWA-mem (v0.7.17). SAM files were converted to BAM format using SAMtools (v1.9).

#### 2.3.3 Post-alignment processing

Aligned BAM files were validated using Picard (v2.26.9) and processed to sort reads, filter low-quality alignments (MAPQ <20), and mark PCR duplicates using SAMtools and Sambamba (v0.6.6). Tumor purity of each sample was estimated using Sequenza (v3.1.0).

#### 2.3.4 Somatic variant calling

Somatic variants were identified using GATK Mutect2 (v4.2.4.0) with a matched normal sample, a germline resource (gnomAD, hg38), and a panel of normals (locally generated from matched blood DNA). Following variant calling, we applied **FilterMutectCalls** according to GATK Best Practices to minimize false positives. Variants were retained if they met the following criteria: a minimum tumor depth of 10 reads, at least 3 supporting tumor reads, and a minimum allele fraction (VAF) of 0.05. Strand bias was assessed using Fisher's exact test, and variants exhibiting significant strand bias were removed. Variants failing these thresholds were excluded from downstream analyses. Following initial variant calling, variants located on mitochondrial (chrM), unmapped (chr_Un), random (chr_random), and alternative (chr_alt) contigs were manually filtered out prior to downstream analyses.

#### 2.3.5 Variant annotation

Variants were functionally annotated using ANNOVAR (v2020Jun07) against multiple databases: 1000 Genomes, COSMIC, dbSNP, gnomAD, ExAC, and prediction tools like SIFT, PolyPhen-2, CADD, REVEL, and others.

#### 2.3.6 Somatic variant analysis and visualization

We added functional analyses to better understand where these variants occur in proteins, and how they influence cancer-related pathways which are known to drive bladder tumor development. Annotated Variant Call Format (VCF) files were analyzed using Maftools (v3.4.1) in R (v4.0.2). Summary plots, oncoplots, PFAM domain analyses, rainfall plots, Ti/Tv ratios, and pathway enrichment (oncogenic and MSigDB hallmark pathways) were generated. Oncodrive analysis used maftools' oncodrive (Oncodrive CLUST, v2.24.0) with AACol = "aaChange", minMut = 5, and pvalMethod = "zscore" for protein clustering bias. Z-scores compare observed mutation clusters (11-AA window, background from synonymous rates) to uniform expectation; p-values from normal distribution. Multiple-testing adjusted via Benjamini-Hochberg FDR; cross-validated with dNdScv due to small cohort. Intratumoral heterogeneity was quantified using the Mutant-Allele Tumor Heterogeneity (MATH) score, calculated via the plotVaf function in maftools. The MATH score was defined as the ratio of the median absolute deviation (MAD) to the median of the variant allele frequency (VAF) distribution for each sample. To infer tumor clonality and sub-clonal architecture, VAF clustering was performed using the inferHeterogeneity function in maftools, which utilizes a density-based finite mixture model to group variants into distinct clonal clusters.

Tumor Mutational Burden (TMB) was defined as the total number of non-synonymous somatic mutations per megabase (Mb) of the captured exome.

For comparative analysis, the TMB distribution of our cohort was benchmarked against established TCGA bladder cancer (BLCA) datasets using the tcgaCompare function within the maftools R package. To identify disrupted biological processes, mutated genes were mapped to ten canonical oncogenic signaling pathways using the plotPathways function. This analysis calculated the fraction of mutated genes within each pathway compared to the total number of genes associated with that pathway in the TCGA cohorts.

To investigate the relationships between mutated genes, we performed a somatic interaction analysis to identify pairs of genes exhibiting significant co-occurrence or mutual exclusivity. This analysis was conducted using the somaticInteractions function from the maftools R package. Statistical significance for gene-pair associations was determined using a pair-wise Fisher's exact test, with a p-value threshold of <0.05 considered statistically significant. The results were visualized as a triangular matrix highlighting the log-odds ratios and significance levels.

To identify localized hypermutation events, or Kataegis, rainfall plots were generated to visualize the inter-mutation distance (IMD) across the genome. Kataegis clusters were formally defined as genomic regions containing six or more consecutive mutations with an average inter-mutation distance of less than or equal to 1000 base pairs. This analysis and the corresponding visualizations were performed using the maftools R package.

#### 2.3.7 Driver gene identification (dNdScv)

Non-silent somatic mutations were extracted from the MAF object and formatted for dNdScv (v1.2.0 (Martincorena et al., 2017). The analysis was performed using the built-in hg38 reference dataset (refdb = "hg38″) with default covariates and max_muts_per_gene_per_sample = 3 to accommodate hypermutation. dN/dS ratios were estimated separately for missense (wmis), nonsense, essential splice-site, and indel mutations were corrected for gene-specific mutational opportunity, trinucleotide context, and local variation in mutation rate. Gene-level significance was assessed using likelihood-ratio tests, and p-values were adjusted genome-wide using the Benjamini–Hochberg false discovery rate (FDR). Genes with qallsubs_cv < 0.05 were classified as candidate drivers; all others were considered passengers.

#### 2.3.8 Mutational signature analysis

Mutational signatures were analyzed to understand mutagenic processes influencing these tumors and to complement our variant-level findings. The R package MutationalPatterns with the GRCh38 reference genome (BSgenome.Hsapiens.UCSC.hg38) was used to detect mutational signatures. Single nucleotide variants (SNVs) were first extracted from the VCF files and classified into a 96-trinucleotide mutational matrix based on the substitution type and sequence context. Due to limited sample size, *de novo* signature extraction was bypassed in favor of single-sample decomposition. Sample mutational profiles were reconstructed using a strict refitting approach against known COSMIC v3 reference signatures (fit_to_signatures) to quantify the relative contribution of specific mutational processes (e.g., SBS2, SBS3). The stability of these signature attributions was evaluated using 1000 bootstrap iterations. Additionally, cosine similarity matrices were calculated to assess the correlation between sample profiles and reference signatures.

#### 2.3.9 Copy number variation (CNV) analysis

We examined copy number alterations to capture larger genomic changes relevant to bladder cancer biology. CNV detection was performed using CNVkit (v0.9.9). Log2 copy ratios were computed for both on- and off-target reads, before visualizing segment level copy number through Circular Binary Segmentation Log2 ratio thresholds of **±0.3** for copy number loss and **±0.7** for copy number gain were applied, following prior studies for WES-based CNV calling. Per-sample segmentation files and visualization were generated to confirm CNV calls. Tumor purity and ploidy were estimated using Sequenza (v3.1.0), and these metrics were considered when interpreting CNV results [23].

#### 2.3.10 Statistical analysis and visualization

All the statistical analysis and visualization were done using the R programming language (version 4.5.0) in R studio. Using P value of less than 0.05 as a threshold of significance.

## 3 Results

### 3.1 WES of bladder cancer tumor and matched blood sample

The whole-exome sequencing was performed for four sets of biospecimens (blood and tumor tissue) of four individual patients. Median sequencing coverage of target regions in four tumor tissue samples was 65X. The blood samples achieved a median sequencing coverage of 68X. Table-1 describes the available clinical features of the patients.

**Table-1:** Demographic and clinicopathological data of patient 001 to 004. Bladder cancer type (NMIBC or MIBC), histological grade, and tumor stage data were not available for all four patients and therefore has been excluded from the table.

Patient ID	Sex	Age	Cigarette/Betel leaf habit	Hematuria	Histopathology report	Abdominal USG report
001	M	57	Cigarette from age 18, Quit	From Jan ‘20	Low grade Urothelial Carcinoma	N/A
002	M	76	Cigarette habit	From Jul ‘20	N/A	Urinary bladder mass
003	M	65	Cigarette from age 15, Quit	From Oct ‘20	High grade urothelial carcinoma	Moderately enlarged prostate
004	M	75	Cigarette from age 20, Quit	From Jan ‘21	High grade urothelial carcinoma	Vesical mass

MultiQC summary of the sequencing data are shown in Supplementary Figure-1. All FASTQC parameters showed acceptable amount of quality after trimming using trimmomatic.

### 3.2 Somatic mutation statistics

GATK Mutect2 returned 15559 somatic variants (Median count = 3632) in total (SNV + Indel) from 2340 genes in 4 tumor-normal matched samples, Filtering variants present in random (chr_random), alternative (chr_alt), unmapped (chr_Un) and mitochondrial (chrM) chromosomes reduced the final number to 14,131 variants. Among these, 4116 variants were in the exonic region (27% of the total), 7393 variants were in the intronic region and 1783 variants were intergenic. Additionally, 776 variants were in either 3’ or 5’ UTRs and 63 variants were located inside splicing regions. Of the 4116 variants in the exonic region, 2651 were nonsynonymous SNVs (nsSNVs) and 1193 were synonymous. As for the insertion-deletion mutations, 32 were frameshift insertions and 77 were frameshift deletions. 163 exonic variants were found to be either stopgain or startloss mutations. Patient 002 had the highest mutation load.

The number of variants in differently filtered datasets is depicted in Figure-1B. Mutation distribution across chromosomes (Kataegis analysis) for each sample is visualized as a rainfall plot. Chromosome 1, 6 and 16 were regularly detected to carry localized hypermutations indicated by the arrows (Supplementary Figure-2).

**Fig. 1:**
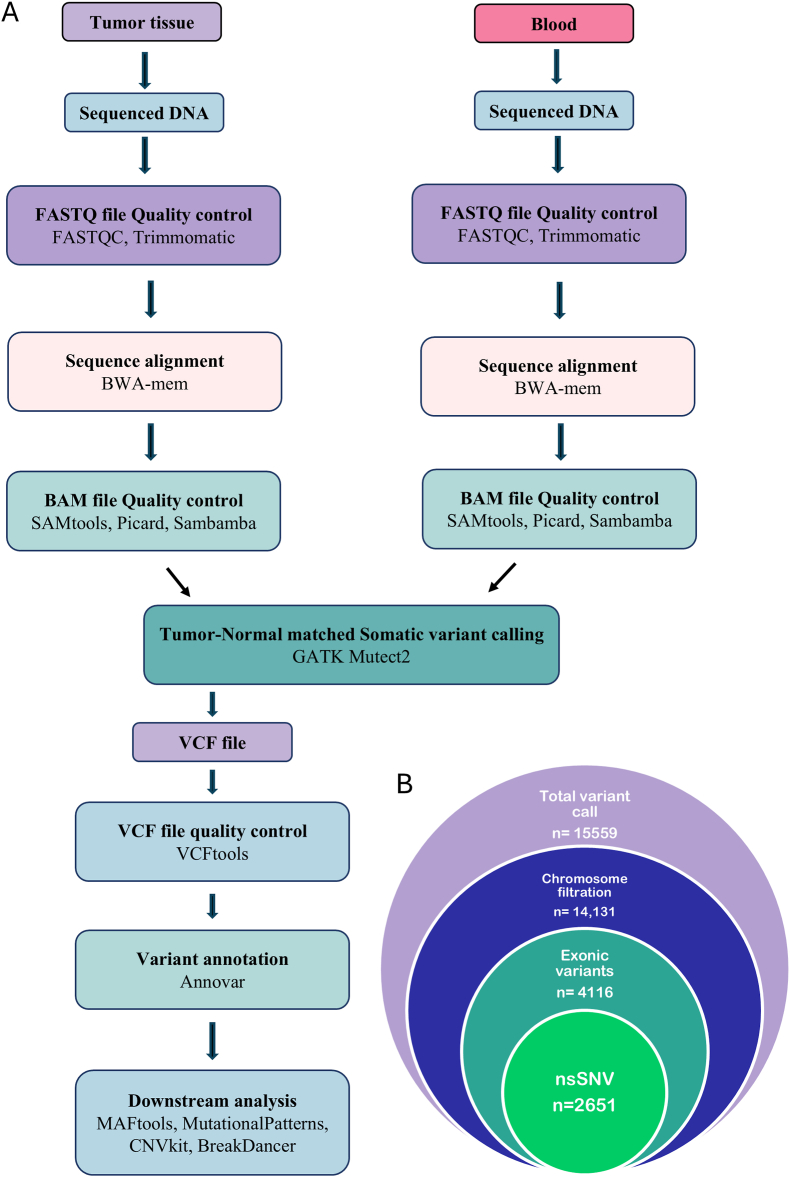
**Overall workflow** (A) Flow-chart displaying overall workflow from raw sequenced file to annotation (B) Venn diagram of number of mutations detected.

Tumor mutation burden (TMB) comparison revealed that the study cohort had higher mutation burden than most of the TCGA patient cohorts. Two of our samples had a higher mutation count than average. Notably the samples had higher mutation load than the lungs cancer (LUAD) cohort and lungs cancer squamous cell carcinoma (LUSC) cohort. However, our study subjects carried less mutation load than skin cancer (SKCM). Mutation burden comparisons with TCGA should be interpreted cautiously, as differences in sequencing platforms and variant calling pipelines may introduce bias. We normalized TMB per megabase; however, direct comparisons remain approximate. (Supplementary Figure-3).

### 3.3 Mutation types and top mutated genes

Maftools found that missense mutations made up the majority of variants in all four samples, followed by in frame deletions and frame shift deletions. Nonsense mutations made up a very tiny proportions of the variants identified (Fig. 2A & E).A majority of all the identified variants were single nucleotide changes. About 500 mutations were identified as deletions, while insertion were low (Fig. 2B). The most frequent nucleotide change was C > T followed by T > C (Fig. 2C). DU_002 sample had the highest number of mutations while the other three samples had similar mutation burden to one another (Fig. 2D). *MUC3A* had the highest number of mutations with 217, a majority of which were missense mutations. *MUC16* was also mutated in all 4 samples with more than 30 distinct mutated sites. Other top mutated genes are shown in Fig. 2F. Ti-Tv analysis showed the samples had higher number of transition mutation compared to transversion mutation (Fig. 3B). To distinguish drivers from length-related passengers in this hypermutated cohort, we applied dNdScv v1.2.0 (Martincorena et al., 2017) using the hg38 reference (refdb = "hg38″), default covariates, and a cap of three mutations per gene per sample. Genome-wide significance was assessed by likelihood-ratio tests with Benjamini–Hochberg FDR correction. Based strictly on the q < 0.05 threshold, MUC3A (q = 0.019), MUC16 (q = 0.023) and AHNAK2 (q = 0.023) met the nominal statistical threshold for a candidate driver genes (Table 2). However, these algorithmic classifications require external validation due to the limited size of the cohort. In addition, the limited size of the pilot cohort (n = 4) severely limits the background mutation rate normalization of the dNdScv model meaning the findings are likely to be artifacts.

**Fig. 2:**
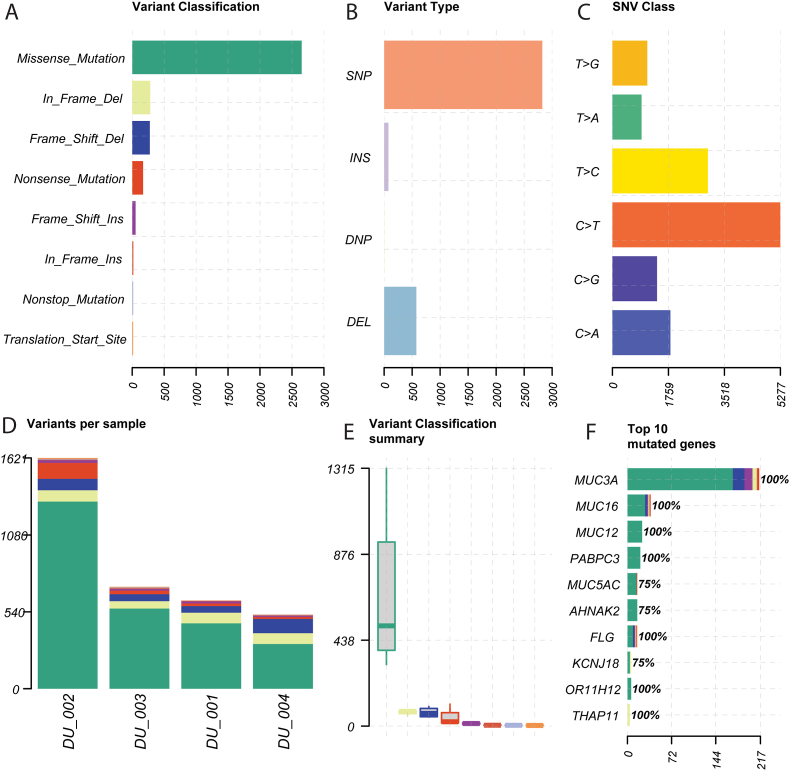
**Maftools summary plot of four samples.** (A) Classification of somatic mutations based on effect (B) Variant types (C) Single nucleotide variation classes (D) Variant distribution based across samples (E) Variant classification summary (F) Variant distribution across top mutated genes.

**Fig. 3:**
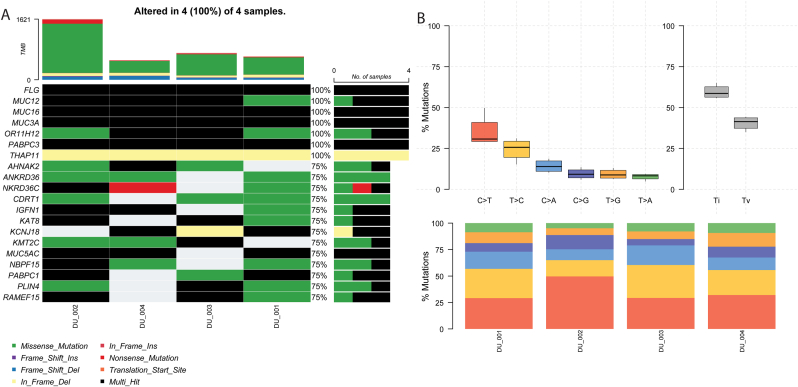
**Mutation effect analysis.** (A) Waterfall plot (Oncoplot) of mutation distribution found in our tumor samples. Each colored cell indicates different mutation types, with black cells meaning a multi-hit for a gene which translates into having more than one mutation in a gene in the same sample. (B) TiTv (Transition-Transversion) plot showing prevalence of transition mutations, especially between C and T nucleotides compared to other SNV classes. The barplot below it displayed the overall distribution of these SNV classes.

**Table 2:** dNdScv results for selected large genes after length correction.

Gene	n_missense	n_indel	wmis_cv	q allsubs_cv	Classification
MUC3A	10	1	3091	0.019	Candidate driver
MUC16	7	3	497	0.023	Candidate driver
AHNAK2	5	0	874	0.023	Candidate driver

* Genes with q value < 0.05 were regarded as driver mutations. Given the large size of these genes and the small cohort (n = 4), their biological validity as a true driver is highly limited. Also note that the mutation counts in Table 2 reflect the dNdScv algorithmic cap of three mutations per sample, differing from the raw counts mentioned above.

Mutation impact analysis of the top mutated genes revealed *FLG* as the most impacted gene followed by *MUC12, MUC16, MUC3A, OR11H12* and *PABPC3* showing multiple types of mutations. All of these genes were mutated in every sample (100%, 4/4). *KMT2C* was mutated in 3 of the 4 patients (75%, 3/4), Two of these patients had confirmed high-grade carcinoma, while the grade for the third (Patient 002) was unavailable, while the single low-grade case (Patient DU_001) had no mutation in this gene. Similarly, *AHNAK2* also showed mutations in 75% (3/4) of patients (Fig. 3A).

**Fig. 4:**
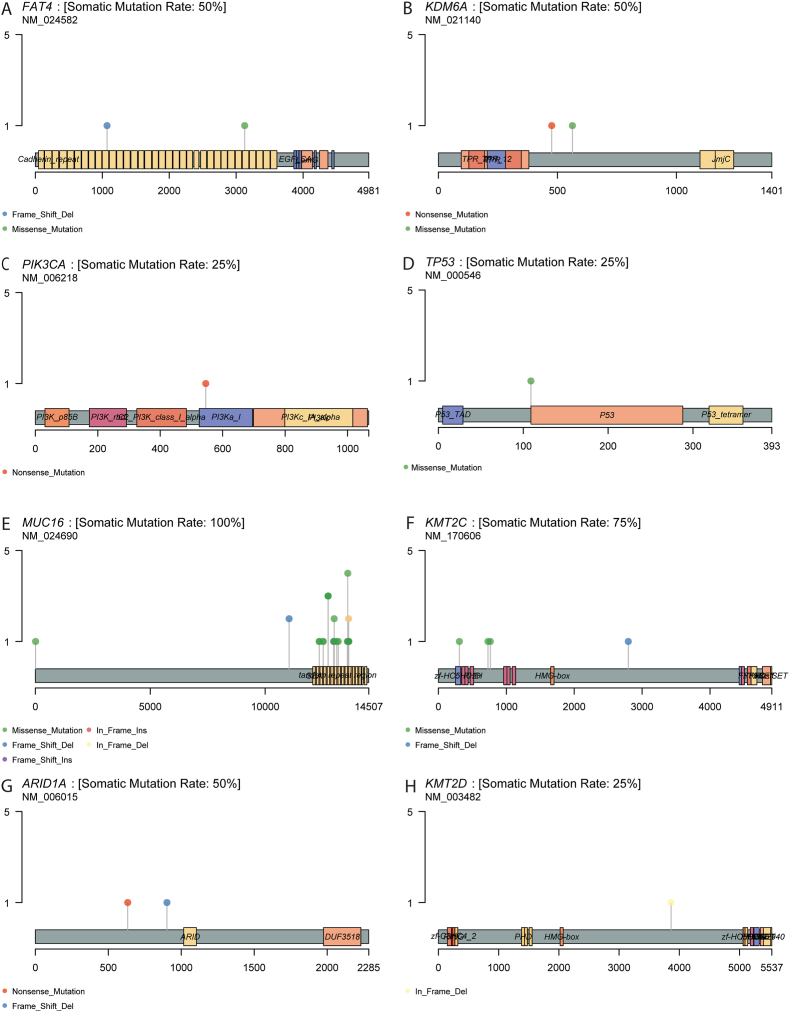
**Lollipop plots** showing GATK Mutect2 detected somatic variants in multiple genes. Displayed genes were considered based on top mutated genes in various bladder cancer studies, gathered from cBioPortal website (A-H) (https://www.cbioportal.org/).

**Fig. 5:**
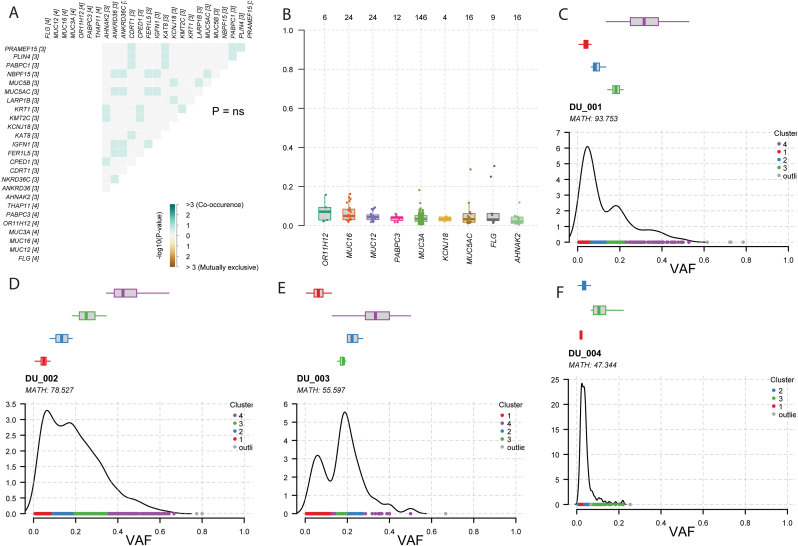
**Distribution of Variant allele frequency.** (A) Co-occurrence and mutual exclusivity of mutations are depicted in the matrix, with statistical significance indicated (*P*= Not Significant; color-coded by –log_10_(P-value)). (B) Comparison of variant allele frequency of top mutated genes. (C–F) Each panel shows raw variant allele frequency (VAF) density distributions grouped into variant clusters, alongside MATH (Mutant-Allele Tumor Heterogeneity) scores. Note that due to low tumor purity, distinct clusters cannot be definitively labeled as subclonal.

Subsequently, top mutated genes from TCGA that are commonly found in bladder cancer were checked manually. *FAT4* and *KDM6A* were mutated in 50% (2/4) of the patients while *PIK3CA* and *TP53* mutation were found in only 25% (1/4) samples. *ARID1A* and *KMT2D* were also mutated in 50% (2/4) and 25% (1/4) cases respectively (Fig. 4A–H). Mutation co-occurrence test showed moderate correlation between several genes such as *KMT2C* and *AHNAK2*, although none was statistically significant (Fig. 5A).

### 3.4 Variant allele frequency analysis

The variant allele frequency (VAF) of top mutated genes were all less than 0.2 meaning less than 20% of the reads had the mutant allele (Fig. 5 B). Clustering analysis of raw VAF grouped the variants into multiple density peaks indicating varying allele distributions among samples (Fig. 5C–F). However, Sequenza analysis revealed that the overall tumor purity of these tissues was notably low, ranging from 10% to 28% (Supplementary Table 2). Because raw VAF is heavily confounded by normal cell admixture, a fully clonal heterozygous mutation in a 20% pure tumor will present with a VAF of approximately 0.10. Therefore, without explicit copy-number and purity-adjusted Cancer Cell Fraction (CCF) modeling, we cannot definitively assign these density clusters to true clonal or sub-clonal populations.

### 3.5 Localized hypermutations of genomic regions

**Fig. 6:**
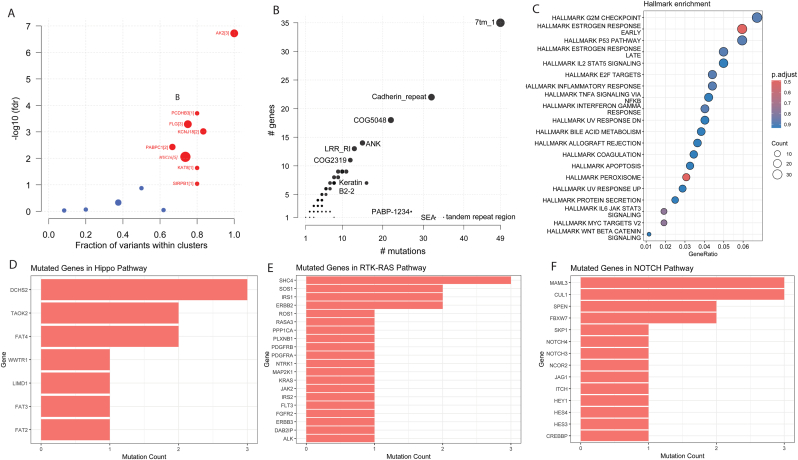
**Mutation clustering and overall pattern** (A) Scatter plot visualizing clustered variations in genes. The size of the points is proportional to clusters found in individual genes. X-axis displays the fraction of mutations observed in the clusters while the Y-axis shows the negative logarithm scale of FDR (False discovery ratio). (B) Protein domains showing highest mutation counts in our study patients. X-axis holds the number of mutations while Y-axis holds the number of mutated genes for each of the domains. Top 5 mutated domains were displayed. (C) Dot plot of cancer hallmark pathway enrichment. Point sizes were based on count and colors were given according to adjusted p-value. (D-F) Number of mutated genes in the top affected pathways.

To investigate whether specific genes harbored non-randomly distributed mutations suggestive of functional importance, we performed a mutational clustering analysis on the bladder cancer whole-exome sequencing dataset. Several genes exhibited significant clustering of mutations, suggesting the potential presence of mutational hotspots rather than randomly distributed passenger mutations. Among these, *AK2, FLG, KCNJ18, PCDHB3, MUC16, PABPC1, KAT8*, and *SIRPB1*met the nominal statistical thresholds in our preliminary analysis. Notably, *AK2* displayed the most prominent signal, with nearly all observed variants falling within three clustered regions and yielding a low nominal false discovery rate (FDR < 1e-6), though this must be interpreted cautiously in a pilot cohort.*MUC16* and *FLG*, two of the most frequently mutated genes in epithelial cancers, also demonstrated clustered mutational patterns, suggesting potential functional relevance beyond background passenger mutation events typically associated with their large genomic sizes. Additional genes such as *KCNJ18* and *PCDHB3* are less well-characterized in bladder cancer but demonstrated reproducible clustering, highlighting them as candidates for further investigation in larger population studies. Similarly, *PABPC1, KAT8*, and *SIRPB1* showed evidence of clustered variants, warranting further investigation into their roles in tumorigenesis (Fig. 6A).

### 3.6 Detecting commonly mutated protein domains

The Pfam domain function from the Maftools package on R was used for domain specific mutation analysis (Fig. 6B). Notably, the 7tm_1 domain, associated with G-protein-coupled receptors (GPCRs), exhibited the highest mutation load (∼50 mutations) and gene count (∼35 genes), suggesting its potential role as a mutational hotspot in bladder cancer.

Other domains with substantial mutation and gene counts included Cadherin_repeat (∼30 mutations, ∼20 genes), COG5048 (∼25 mutations, ∼17 genes), and *ANK* (Ankyrin repeats) (∼20 mutations, ∼12 genes). COG5048 is a member of the zinc-finger protein family, and its function includes DNA recognition [24], transcriptional activation and regulation of apoptosis. 7tm_1 protein domain is a part of the rhodopsin superfamily of proteins and is responsible for cell signaling. Cadherin repeats domains occur in the extracellular regions and are thought to mediate cell contacts [25]. These domains are known to be involved in critical cellular processes such as cell adhesion, signaling, and protein-protein interactions, implicating their disruption in tumorigenesis [25]. Conversely, domains such as PABP-1234+ and SEA-tandem repeat region showed low mutation and gene counts, possibly reflecting more specialized or less frequently altered regions in bladder cancer.

### 3.7 Affected signaling pathways and cancer hallmark enrichment

Mutation affected pathways included *Hippo, NOTCH, RTK-RAS,* and *WNT* in all four samples (100%, 4/4) while the TGF-Beta pathway was affected in 50% (2/4) of patients. *RTK-RAS* and *NOTCH* were the two most affected pathways. About 22% of all genes in these two pathways were mutated (34 out of 156 genes). *RTK-RAS* pathway is one of the most frequently mutated pathways in cancer while the *NOTCH* pathway is a regulator of self-renewal and differentiation in several tissues and cell types and is also a binary cell-fate determinant. The mutated genes of the top three impacted pathways the shown in Fig. 6D-F. Hallmark enrichment of the affected genes showed G2M checkpoint, estrogen response and p53 related mutations affected in all four samples (Fig. 6C).

### 3.8 Detection of mutational signatures

**Fig. 7:**
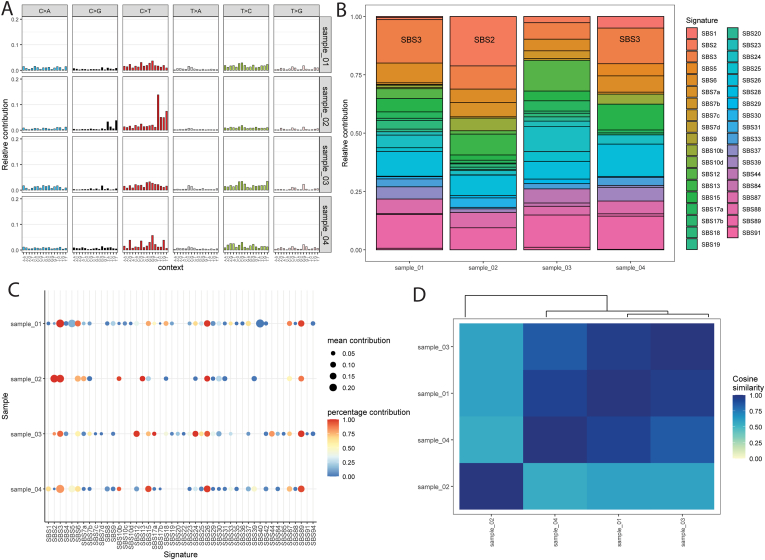
**Single sample decomposition analysis of bladdes cancer whole exome sequencing using COSMIC signatures.** (A) 96 profile plot showing relative contribution of type of mutation and signatures per sample. (B) Relative contribution of different COSMIC signatures per sample before bootstrapping. (C) Dot plots showing contribution mean contribution and relative contribution after bootstrapping. (D) Heatmap showing Cosine similarities between samples.

Mutational signature analysis, performed by decomposing sample profiles against the COSMIC v3 reference database, identified two dominant mutational processes within the cohort. Samples DU_001, DU_003, and DU_004 were primarily characterized by signature SBS3, a pattern associated with defects in homologous recombination based DNA repair. In contrast, sample DU_002 displayed a distinct mutational profile dominated by SBS2, a signature linked to the aberrant activity of the AID/APOBEC family of cytidine deaminases (see Fig. 7A-D).

### 3.9 Detection of copy number variations (CNVs)

**Fig. 8:**
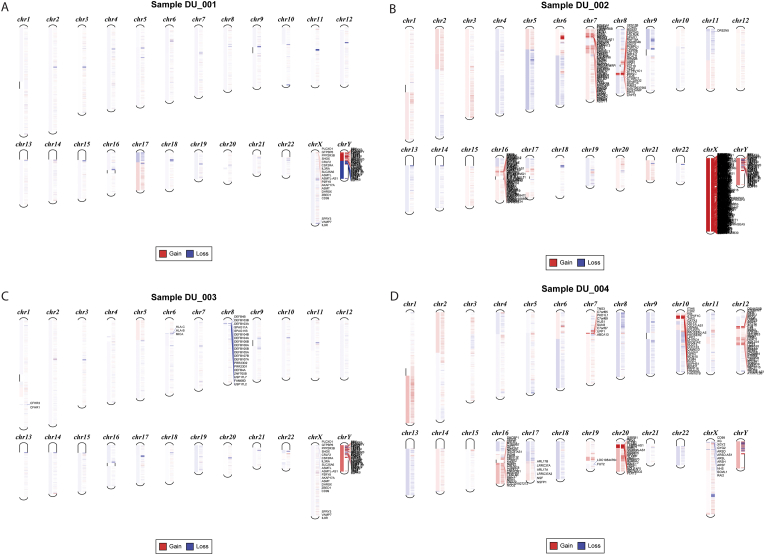
**Chromosomes of individual patients showing respective copy number alterations.** Copy number ratio threshold for A and C were The log-ratio thresholds (0.3 for loss, 0.7 for gain) were chosen based on CNVkit guidelines for WES data and validated in previous studies to balance sensitivity and specificity. Minimum probe limit was kept at 3.

Analysis of somatic CNVs identified chromosomal loss in regions 1p12 (containing *NOTCH2*), 5q12, 8p11.22, 9p21.2-p21.3, 12p13.31 (containing *APOBEC1*), 18q11.1, Xq23 (containing *AGTR2*), Xq28 and Yp11.3 and chromosomal gain in regions 1q21.3, 6p22.3 (containing *DEK* and *E2F3*), 7p14.1, 7p21.1, 10p14 and 19q13.2 (containing *RYR1*). These losses and gains in individual tumor samples are shown in Figure-8(A-D). Notably, Sample DU_001 showed loss in Y chromosome (25%, 1/4), a phenomenon recently described in bladder cancer. It is to be noted that the observed Y-chromosome deletion was identified based on CNVkit segmentation and confirmed across multiple bins; however, given the small cohort and technical limitations of WES-based CNV detection, this finding should be considered preliminary and requires validation using orthogonal methods (e.g., qPCR or SNP arrays) to exclude artifacts.

## 4 Discussion

The search for noninvasive methods for the detection of bladder cancer has been long pursued is largely dependent on discovering common and recurrent somatic mutations in a population so they can be used as biomarkers for cancers detection. With this in mind, we attempted to detect common somatic mutations in Bladder cancer patients through Next generation sequencing using the Illumina Hiseq platform to exome-sequence four tumor samples. Despite the limited sample size, this is, to our knowledge, the first WES-based exploratory study of bladder cancer patients in the Bangladeshi population, providing an important foundation for future genomic investigations.

[26] [27]GATK Mutect2 variant caller has a mode where alongside tumor and matched normal BAM files, Panel of Normals (PoN) file and germline resource file could also be provided [28]. These additional files were given as input to help the existing matched normal file to detect only credible somatic variants. PoN files contain DNA sequences from healthy samples only [26]. Therefore, these files do not contain any somatic variants and neither does the germline resource file, which contains only the germline variants. Including these corrective files result in results with much less noise [27]. According to GATK Broad Institute, PoN files have another purpose of capturing recurrent technical artifacts to improve variant calling results. For a better understanding of called variants, variant annotation is a well-recognized strategy. Annotation enables the creation of clinical reports by mining information from multiple publicly available databases [29]. [30].

We found somatic mutations in *FAT4, KDM6A, PIK3CA, ARID1A, KMT2D, KMT2C and AHNAK2*. *KDM6A, ARID1A, KMT2C and KMT2D* are epigenetic regulators, while *FAT4* and *PIK3CA* genes are signaling mediators involved in oncogenic pathways [31]. These are the top mutated genes in Bladder cancer studies according to cBioPortal [32]. Other frequently mutated genes were *MUC12* and *FLG*. The *KMT2C* gene was found to be mutated in a study on different immunotypes of MIBC [33]. *FLG* is a relatively new gene to be found mutated in Bladder cancer patients and so are *MUC3A, KCNJ18* and *AHNAK2*. On the other hand, study patients had the most missense mutations as Single Nucleotide Polymorphisms (SNPs) and a frequent C > T conversion, similar to other studies [30]. *AHNAK2* gene works in the regulation of RNA splicing. One proteomics study of *AHNAK2* regarded this gene as a potential biomarker for bladder cancer, which can differentiate between urocystitis and low-grade carcinoma with invasive high-grade [34]. Another study found *AHNAK2* to be associated with aggressive pathological findings obtained by radial cystectomy and also with worse recurrence-free survival (RFS) and cancer-specific survival (CSS) [35]. *TP53*, a key tumor suppressor, regulates cell cycle and DNA repair; its mutation leads to genomic instability and uncontrolled proliferation [36]. Epigenetic regulators such as *KDM6A, ARID1A, KMT2C*, and *KMT2D* influence chromatin remodeling and transcriptional regulation, and their disruption may promote tumor heterogeneity and progression [37] [38]. *PIK3CA* mutations activate the *PI3K/AKT* signaling pathway, driving cell survival and growth, while *FAT4*, a component of the Hippo pathway, is implicated in cell polarity and contact inhibition [39] [40]. Frequent mutations in mucin genes (*MUC16, MUC3A, FLG*) could alter cell adhesion and immune evasion, contributing to aggressive phenotypes [41]. Notably, *AHNAK2*, associated with epithelial-mesenchymal transition and poor prognosis in other cancers, may play a role in invasion and metastasis in bladder cancer [42]. These alterations collectively affect critical pathways including cell cycle control, chromatin dynamics, and oncogenic signaling, underscoring their potential functional relevance. *KMT2C* mutations were restricted to high-grade tumors (3/3) and absent in the single low-grade case, suggesting a possible grade association, while *TP53* showed no consistent clinical pattern and occurred in only one patient. *FLG* alterations were detected across all cases, indicating no discriminatory association with grade, smoking history, or hematuria. Because all patients were smokers and presented with hematuria, meaningful molecular–clinical contrasts were not possible, underscoring the need for larger, better-stratified cohorts.

Given the small sample size (n = 4), mutation frequency estimates in these results should be interpreted with caution. For example, a mutation observed in two samples corresponds to an estimated frequency of 50%, but the 95% confidence interval is wide (approximately 15%–85%). This reflects limited statistical power to detect rare variants and accurately characterize population-level mutation prevalence. Moreover, comparison with TCGA data on bladder cancer acquired from cBioPortal showed reduced mutation frequency of genes like TP53 and higher frequency for *MUC16* and *TTN* (Supplementary Table- 3). Future studies with larger cohorts are essential to refine these estimates and validate observed trends.

Kataegis phenomenon is described as a pattern of localized hypermutations identified in some cancer genomes. This is a phenomenon where a large number of highly patterned mutations occur in a small region of DNA. Cancer genomes and exomes sequenced by the collective effort of different groups as well as TCGA and ICGC have enabled researchers to assess the consequences of hypermutation in cancer development [43]. [44]Chromosome 1, 6 and 16 were regularly detected to carry localized hypermutations in our study patients. This can mean that these chromosomes and the genes they carry are more prone to somatic mutations in Bladder cancer patients than in other cancer types. These particular chromosomes can face breakage and other chromosomal aberrations and genes residing in these genes can be susceptible to gene fusions. Together, these preliminary results generate the hypothesis that these tumors may harbor localized mutation clustering, pointing to potential driver events that warrant further study. The identification of both known cancer-associated genes (e.g., *MUC16*) and less-characterized candidates (e.g., *AK2, KAT8*) underscores the potential of clustering-based analyses to uncover genomic contributors to bladder cancer pathobiology.

The VAF analysis of top mutated genes revealed that all had allele frequencies below 0.2. While low VAFs are sometimes indicative of sub-clonal architecture, our Sequenza analysis revealed low tumor cellularity across all samples (10%–28%). In the context of heavy normal tissue admixture, even fully clonal founding mutations will present with raw VAFs artificially depressed into the 0.05–0.15 range. Consequently, the distinct VAF clusters (Clusters 1–3 vs. Cluster 4) observed in our analysis cannot be definitively interpreted as sub-clonal diversity versus the primary clonal population. Definitive conclusions regarding intratumoral heterogeneity and late-stage tumor evolution cannot be drawn from raw VAFs alone; future studies must incorporate explicit Cancer Cell Fraction (CCF) modeling that integrates tumor purity and local copy-number states to accurately resolve clonal structures [45].

The median coverage in our study (65 × for tumor and 68 × for normal) is lower than the commonly cited WES standard of ∼100 × . Reduced coverage may limit sensitivity for detecting low-VAF variants and could lead to underestimation of tumor mutational burden (TMB) [46]. While we applied stringent filtering criteria to minimize false positives, these factors should be considered when interpreting our results. Future studies with higher coverage will improve detection of subclonal mutations and refine TMB estimates.

Diverse mutational processes generate clustered single-nucleotide changes in human somatic cells [47]. These clustered mutations are common in cancer genomes, and they have a significant influence on changing the overall survival of patients [48]. Clustering mutations has been associated with many events such as heterogeneous mutation rates across the genome, biophysical characteristics of exogenous carcinogens and large mutational events related to genome instability [48]. Adenylate kinase 2 (AK2) showed the highest level of clustered mutation. AK2, implicated in adenine nucleotide synthesis has been shown to have impact on cell death and proliferation [44]. Another clustered mutation was the *PCDHB3* mutation. The gene is a part of the protocadherin family, and several studies found association on this gene with lung, colorectal and gastric cancer [49] [50].

*KCNJ18* clustered mutation, however, was observed in half of the study patients. The clustered mutation was seen in the *MUC16* gene as well. Mutation clusters might have a positive correlation with higher TMB and so further investigations with large-scale clinically annotated whole-genome sequenced cancers are required to fully understand the clinical implications of clustered mutations.

Studying protein domain mutations can help increase the statistical power of detecting recurrent mutations by extending the notion from genes to homologous protein domains. As gene functions depend on particular protein domains, somatic mutation events in these domains can be helpful in the interpretation of those mutations in terms of functional changes occurring in genes themselves [51]. From our analysis, it was seen that 7tm_1 and cadherin repeat domains were the most mutated and most genes affecting domain, respectively. 7tm_1 domain are the core of GPCR signaling modules whose mutations can alter growth, invasion, and other cancer-related signaling cascades central to tumor biology [52]. Cadherin repeat domains mediate calcium-dependent cell-cell adhesion, and their disruption promotes loss of tissue architecture and enhances tumor progression and metastasis [53]. In bladder cancer, *CDH1* mutations truncate its extracellular cadherin repeats, especially in plasmacytoid carcinoma, leading to loss of cell–cell adhesion and aggressive invasion. Likewise, *FAT1* mutations frequently remove large portions of its 34 cadherin repeats, disabling adhesion and tumor-suppressive signaling in muscle-invasive disease and 7tm_1 works in cell signaling. A large number of mutations in these two protein domains can result in the ceasing of regular cell function.

Multiple signaling pathways have already been investigated in the process of the pathogenesis of Bladder cancer. *RAS* pathway mutation was found in 85% of low-grade NMIBC cases in one study [54]. *NOTCH* signaling suppresses tumor growth and proliferation and mutations in *NOTCH1* and *NOTCH2* genes result in the loss of function of this pathway [55]. Increased aggressiveness of bladder cancer and worsened prognosis was observed in patients with low *NOTCH* signaling activity as well. Another signaling pathway, *Hippo*, is an important regulator of tissue homeostasis and cell growth. *TAZ* gene of this pathway is a crucial regulator in various human cancers. This pathway was found to affect the proliferation, metastasis, and drug resistance of Bladder cancer [56]. A host of oncogenes and tumor suppressor genes of these pathways were found to be mutated in our study patients, suggesting these pathways do affect Bladder cancer progression. Selected genes from these pathways were observed to play role in cancer hallmark pathways as well, proving their contribution to Bladder cancer.[63][64][65]

The identification of distinct mutational signatures highlights divergent underlying oncogenic mechanisms within the cohort. The prevalence of SBS3 suggests a "BRCAness" phenotype in the majority of samples, indicating a failure in double-strand break repair that may render these tumors susceptible to therapeutic strategies targeting homologous recombination deficiency, such as PARP inhibitors or platinum-based chemotherapy [57]. Conversely, the presence of SBS2 in DU_002 points to an etiology driven by active enzymatic mutagenesis rather than repair deficiency, likely stemming from APOBEC enzyme hyperactivity on single-stranded DNA during replication stress [58]. These findings generate the hypothesis that despite a shared diagnosis, these tumors may evolved through divergent pathways, one driven by genomic instability and the other by endogenous enzymatic hypermutation.

Copy number variation analysis resulted in the detection of gain and loss in multiple genes responsible for normal cell signaling and proliferation. *NOTCH2* gene regulates cell-fate determination, *APOBEC1* is involved in posttranscriptional editing and also in the regulation of gene expression, *AGTR2* works in inhibition of cell proliferation, *DEK* gene is involved in chromatin organization while *E2F3* gene is a transcription activator. Especially in patient DU_001 significant gene loss was observed in the Y chromosome marking it the first such case in Bangladeshi population [59]. However, CNV detection from WES data can be affected by coverage variability and tumor purity. While matched controls and purity estimation were used, genome-wide reliability, particularly for sex chromosomes, requires further validation. A low number of sequenced samples and lack of validation of called mutations in other patients are the main limitations of this study. Nevertheless, this is a novel project for the Bangladeshi population and will act as a foundation for future projects. Novel treatment strategies can be developed based on findings like localized hypermutation, commonly mutated protein domains, mutated signaling pathways and mutation signatures after the findings in this paper have been validated in vitro and in vivo. Further comprehensive studies are necessary beforehand to build up a significant mutation panel which can then be looked into to elect biomarkers and utilize them in modern, rapid, and convenient diagnostic procedures.

## 5 Conclusion

This is the first WES-based pilot study in Bangladeshi bladder cancer patients, serving as a hypothesis-generating foundation to explore potential somatic mutations in this population. While we identified some common mutations, rare mutations were also identified. A key limitation to our study is the small sample size and a sequencing depth of ∼65X, which is why experimental validation, expanded sample size, and functional assays are recommended for future studies. Our study lays the foundation for these further studies to characterize the heterogeneous nature of the bladder cancer genome.

Beyond providing preliminary genomic insights, this study has translational implications for bladder cancer management in Bangladesh. Future work should focus on validating these candidate biomarkers in larger, ethnically diverse cohorts and integrating functional assays to establish their clinical relevance. Such efforts will pave the way for precision oncology approaches tailored to this population.

## Funding

We acknowledge the Centennial Research Grant (CRG) program of the 10.13039/501100006523University of Dhaka for funding this study.

## CRediT authorship contribution statement

**Hasib Uddin Ahmed:** Data curation, Formal analysis, Investigation, Methodology, Visualization, Writing – original draft, Writing – review & editing. **Md Raiyan Hosen:** Data curation, Formal analysis, Investigation, Methodology, Validation, Visualization, Writing – original draft, Writing – review & editing. **Munshi Akid Mostofa:** Data curation, Investigation, Methodology, Project administration, Resources, Writing – review & editing. **Md Akmal Hossain:** Data curation, Investigation, Project administration, Resources, Writing – review & editing. **Md Omar Faruk:** Investigation, Resources, Supervision, Validation, Writing – original draft, Writing – review & editing. **Hossain Uddin Shekhar:** Data curation, Funding acquisition, Investigation, Resources, Supervision, Writing – review & editing. **AKM Mahbub Hasan:** Conceptualization, Funding acquisition, Project administration, Supervision, Writing – review & editing. **Md Ismail Hosen:** Conceptualization, Funding acquisition, Investigation, Methodology, Project administration, Resources, Software, Supervision, Writing – original draft, Writing – review & editing.

## Declaration of competing interest

Authors declare no conflict of interests that could influence or bias the work.

## Figures and Tables

**figs1:**
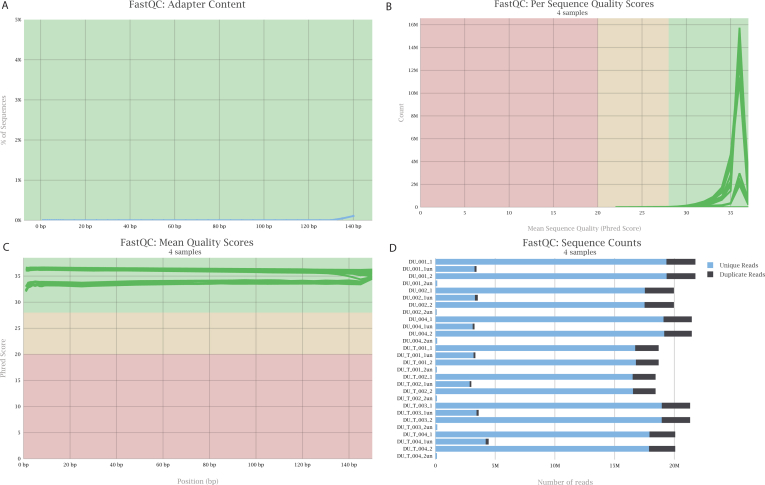


**figs2:**
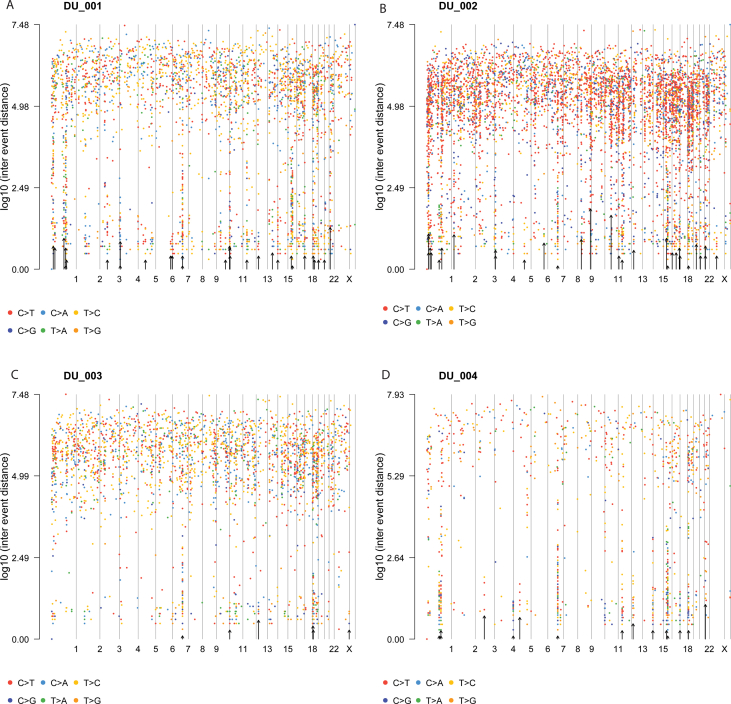


**figs3:**
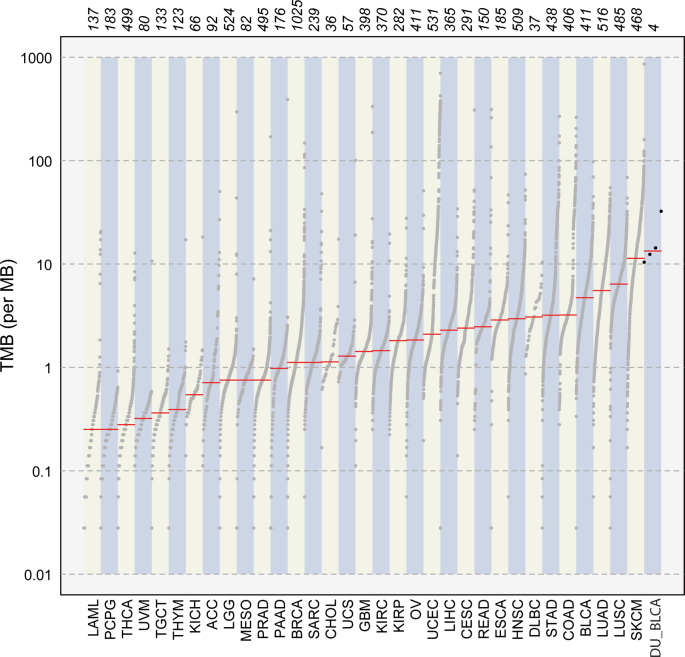


## Data Availability

Data will be made available on request.

## References

[1] Bray F., Laversanne M., Sung H., Ferlay J., Siegel R.L., Soerjomataram I. (2024). Global cancer statistics 2022: GLOBOCAN estimates of incidence and mortality worldwide for 36 cancers in 185 countries. CA Cancer J. Clin..

[2] Leal J., Luengo-Fernandez R., Sullivan R., Witjes J.A. (2016). Economic burden of bladder cancer across the European Union. Eur. Urol..

[3] Alexandrov L.B., Nik-Zainal S., Wedge D.C., Aparicio S.A.J.R., Behjati S., Biankin A.V. (2013). Signatures of mutational processes in human cancer. Nature.

[4] World Health Organization (2022). https://gco.iarc.who.int/media/globocan/factsheets/populations/50-bangladesh-fact-sheet.pdf.

[5] Freedman N.D., Silverman D.T., Hollenbeck A.R., Schatzkin A., Abnet C.C. (2011). Association between smoking and risk of bladder cancer among men and women. JAMA.

[6] Sylvester R.J., Rodríguez O., Hernández V., Turturica D., Bauerová L., Bruins H.M. (2021). European Association of urology (EAU) prognostic factor risk groups for non–muscle-invasive bladder cancer (NMIBC) incorporating the WHO 2004/2016 and WHO 1973 classification systems for grade: an update from the EAU NMIBC guidelines panel. Eur. Urol..

[7] Kirkali Z., Chan T., Manoharan M., Algaba F., Busch C., Cheng L. (2005). Bladder cancer: epidemiology, staging and grading, and diagnosis. Urology.

[8] Wallerand H., Bernhard J.C., Culine S., Ballanger P., Robert G., Reiter R.E. (2011). Targeted therapies in non-muscle-invasive bladder cancer according to the signaling pathways. Urol. Oncol.: Seminars and Original Investigations.

[9] Burger M., Catto J.W.F., Dalbagni G., Grossman H.B., Herr H., Karakiewicz P. (2013). Epidemiology and risk factors of urothelial bladder cancer. Eur. Urol..

[10] Safiri S., Kolahi A.A., Naghavi M., Nejadghaderi S.A., Mansournia M.A., Sullman M.J.M. (2021). Global, regional and national burden of bladder cancer and its attributable risk factors in 204 countries and territories, 1990–2019: a systematic analysis for the global burden of disease study 2019. BMJ Glob. Health.

[11] Shin K., Lim A., Odegaard J.I., Honeycutt J.D., Kawano S., Hsieh M.H. (2014). Cellular origin of bladder neoplasia and tissue dynamics of its progression to invasive carcinoma. Nat. Cell Biol..

[12] Shin J.H., Lim J.S., Jeon B.H. (2018). Bladder Cancer.

[13] Liao Y., Tang H., Wang M., Wang K., Wang Y., Jiang N. (2021). The potential diagnosis role of TP53 mutation in advanced bladder cancer: a meta-analysis. J. Clin. Lab. Anal..

[14] Shariat S.F., Ashfaq R., Sagalowsky A.I., Lotan Y. (2007). Predictive value of cell cycle biomarkers in Nonmuscle invasive bladder transitional cell carcinoma. J. Urol..

[15] Kim J., Akbani R., Creighton C.J., Lerner S.P., Weinstein J.N., Getz G. (2015). Invasive bladder cancer: genomic insights and therapeutic promise. Clin. Cancer Res..

[16] Liu X., Zhang W., Geng D., He J., Zhao Y., Yu L. (2014). Clinical significance of fibroblast growth factor receptor-3 mutations in bladder cancer: a systematic review and meta-analysis. Genet. Mol. Res..

[17] Wu X.R. (2005). Urothelial tumorigenesis: a tale of divergent pathways. Nat. Rev. Cancer.

[18] Forster J.A., Paul A.B., Harnden P., Knowles M.A. (2011). Expression of NRG1 and its receptors in human bladder cancer. Br. J. Cancer.

[19] Zvereva M., Pisarev E., Hosen I., Kisil O., Matskeplishvili S., Kubareva E. (2020). Activating telomerase tert promoter mutations and their application for the detection of bladder cancer. Int. J. Mol. Sci..

[20] Ahmed H.U., Hafiz A., Islam M.M.T., Kabir Y. (2025). Exploring the relationship between genetic polymorphisms and cancer in the Bangladeshi population. J. Bangladesh Acad. Sci..

[21] National Human Genome Research Institute (2021). To sequence the exome or the genome: that is the question. https://www.genome.gov/27555511/to-sequence-the-exome-or-the-genome-that-is-the-question.

[22] Rehm H.L., Bale S.J., Bayrak-Toydemir P., Berg J.S., Brown K.K., Deignan J.L. (2013). ACMG clinical laboratory standards for next-generation sequencing. Genet. Med..

[23] Plagnol V., Curtis J., Epstein M., Mok K.Y., Stebbings E., Grigoriadou S. (2012). A robust model for read count data in exome sequencing experiments and implications for copy number variant calling. Bioinformatics.

[24] Jen J., Wang Y.C. (2016). Zinc finger proteins in cancer progression. J. Biomed. Sci..

[25] Berx G., van Roy F. (2009). Involvement of members of the cadherin superfamily in cancer. Cold Spring Harbor Perspect. Biol..

[26] Lennon N.J., Adalsteinsson V.A., Gabriel S.B. (2016). Technological considerations for genome-guided diagnosis and management of cancer. Genome Med..

[27] De Schaetzen Van Brienen L., Larmuseau M., Van Der Eecken K., De Ryck F., Robbe P., Schuh A. (2020). Comparative analysis of somatic variant calling on matched FF and FFPE WGS samples. BMC Med. Genom..

[28] Alganmi N., Abusamra H. (2023). Evaluation of an optimized germline exomes pipeline using BWA-MEM2 and Dragen-GATK tools. PLoS One.

[29] Dumur C.I. (2014). Available resources and challenges for the clinical annotation of somatic variations. Cancer Cytopathol.

[30] Yang K., Yu W., Liu H., Ding F., Zhang Y., Zhang Y. (2021). Comparison of genomic characterization in upper tract urothelial carcinoma and urothelial carcinoma of the bladder. Oncologist.

[31] Guo C., Chen L.H., Huang Y., Chang C.-C., Wang P., Pirozzi C.J. (2013). KMT2D maintains neoplastic cell proliferation and global histone H3 lysine 4 monomethylation. Oncotarget.

[32] Voutsadakis I.A. (2022). Urothelial bladder carcinomas with high tumor mutation burden have a better prognosis and targetable molecular defects beyond immunotherapies. Curr. Oncol..

[33] Chen Z., Liu G., Liu G., Bolkov M.A., Shinwari K., Tuzankina I.A. (2021). Defining muscle-invasive bladder cancer immunotypes by introducing tumor mutation burden, CD8+ T cells, and molecular subtypes. Hereditas.

[34] Witzke K.E., Großerueschkamp F., Jütte H., Horn M., Roghmann F., von Landenberg N. (2019). Integrated fourier transform infrared imaging and proteomics for identification of a candidate histochemical biomarker in bladder cancer. Am. J. Pathol..

[35] Koguchi D., Matsumoto K., Shimizu Y., Kobayashi M., Hirano S., Ikeda M. (2021). Prognostic impact of ahnak2 expression in patients treated with radical cystectomy. Cancers (Basel).

[36] Hertel A., Storchová Z. (2025). The role of p53 mutations in early and late response to mitotic aberrations. Biomolecules.

[37] Zhang D., Zhao X., Gao Y., Wang M., Xiao M., Zhu K. (2024). Inactivation of KDM6A promotes the progression of colorectal cancer by enhancing the glycolysis. Eur. J. Med. Res..

[38] Hung Y.H., Hsu M.C., Chen L.T., Hung W.C., Pan M.R. (2019). Alteration of epigenetic modifiers in pancreatic cancer and its clinical implication. J. Clin. Med..

[39] Zardavas D., Phillips W.A., Loi S. (2014). PIK3CA mutations in breast cancer: reconciling findings from preclinical and clinical data. Breast Cancer Res..

[40] Willecke M., Hamaratoglu F., Kango-Singh M., Udan R., Chen C lin, Tao C. (2006). The fat cadherin acts through the Hippo tumor-suppressor pathway to regulate tissue size. Curr. Biol..

[41] Zhang X.Y., Hong L.L., Ling Z.Q. (2024 24:1 2024). MUC16: clinical targets with great potential. Clin. Exp. Med..

[42] Liu G., Guo Z., Zhang Q., Liu Z., Zhu D. (2020). AHNAK2 promotes migration, invasion, and epithelial-mesenchymal transition in lung adenocarcinoma cells via the TGF-β/Smad3 pathway. OncoTargets Ther..

[43] Roberts S.A., Gordenin D.A. (2014). Hypermutation in human cancer genomes: footprints and mechanisms. Nat. Rev. Cancer.

[44] Kim H., Jeong M., Na D.H., Ryu S.H., Jeong E Il, Jung K. (2022). AK2 is an AMP-sensing negative regulator of BRAF in tumorigenesis. Cell Death Dis..

[45] Sisoudiya S.D., Tukachinsky H., Keller-Evans R.B., Schrock A.B., Huang R.S.P., Gjoerup O. (2025). Tissue-based genomic profiling of 300,000 tumors highlights the detection of variants with low allele fraction. npj Precis. Oncol..

[46] Makrooni M.A., O'Sullivan B., Seoighe C. (2022). Bias and inconsistency in the estimation of tumour mutation burden. BMC Cancer.

[47] Carlson J., Locke A.E., Flickinger M., Zawistowski M., Levy S., Myers R.M. (2018). Extremely rare variants reveal patterns of germline mutation rate heterogeneity in humans. Nat. Commun..

[48] Bergstrom E.N., Kundu M., Tbeileh N., Alexandrov L.B. (2022). Examining clustered somatic mutations with SigProfilerClusters. Bioinformatics.

[49] Zhou J., Zhan J., Zhang H., Liu L., He Y., Le Y. (2025). Comprehensive evaluation of the PCDHB family in gastric cancer: prognostic significance, immune infiltration, and experimental validation of PCDHB5's oncogenic role. Int. Immunopharmacol..

[50] Ginn L., Maltas J., Baker M.J., Chaturvedi A., Wilson L., Guilbert R. (2023). A TIAM1-TRIM28 complex mediates epigenetic silencing of protocadherins to promote migration of lung cancer cells. Proc. Natl. Acad. Sci. U. S. A..

[51] Miller M.L., Reznik E., Gauthier N.P., Aksoy B.A., Korkut A., Gao J. (2015). Pan-Cancer analysis of mutation hotspots in protein domains. Cell Syst..

[52] Khan I.R., Khurshid S., Almawash S., Kumar R., Akil A.S.A.S., Bhat A.A. (2025). G protein-coupled receptor signaling: implications and therapeutic development advances in cancers. MedComm.

[53] Kaszak I., Witkowska-Piłaszewicz O., Niewiadomska Z., Dworecka-Kaszak B., Toka F.N., Jurka P. (2020). Role of cadherins in Cancer-A review. Int. J. Mol. Sci..

[54] Kompier L.C., Lurkin I., van der Aa M.N.M., van Rhijn B.W.G., van der Kwast T.H., Zwarthoff E.C. (2010). FGFR3, HRAS, KRAS, NRAS AND PIK3CA mutations in bladder cancer and their potential as biomarkers for surveillance and therapy. PLoS One.

[55] Maraver A., Fernandez-Marcos P.J., Cash T.P., Mendez-Pertuz M., Dueñas M., Maietta P. (2015). NOTCH pathway inactivation promotes bladder cancer progression. J. Clin. Investig..

[56] Xia J., Zeng M., Zhu H., Chen X., Weng Z., Li S. (2017). Emerging role of Hippo signalling pathway in bladder cancer. J. Cell Mol. Med..

[57] Polak P., Kim J., Braunstein L.Z., Karlic R., Haradhavala N.J., Tiao G. (2017). A mutational signature reveals alterations underlying deficient homologous recombination repair in breast cancer. Nat. Genet..

[58] Jakobsdottir G.M., Brewer D.S., Cooper C., Green C., Wedge D.C. (2022). APOBEC3 mutational signatures are associated with extensive and diverse genomic instability across multiple tumour types. BMC Biol..

[59] Abdel-Hafiz H.A., Schafer J.M., Chen X., Xiao T., Gauntner T.D., Li Z. (2023). Y chromosome loss in cancer drives growth by evasion of adaptive immunity. Nature.

