# Supplementary material for: Whole exome sequencing identifies somatically mutated genes in bladder cancer: A pilot study from Bangladesh
Source: Biochem Biophys Rep. 2026 Apr 3;46:102574. doi: 10.1016/j.bbrep.2026.102574 (PMC13087579; doi:10.1016/j.bbrep.2026.102574)
Supplement: Multimedia component 5 [file mmc5.docx]

**Appendix A. Supplementary data**

**Supplementary Figure-1: Sequencing quality control report by FASTQC and summarized by multiQC**. (A) Showes the percentage of adapter content (B) Quality score distribution of base pair (C) Mean quality score per base position (D) Sequence counts of paired and unpaired reads showing both unique and duplicate reads

**Supplementary Figure-2: Rainfall plots displaying SNV calls as dots separated by chromosome numbers to detect localized hypermutations.** Each dot depicts a SNV and is color-coded according to the six substitution classes shown below the plots. The genomic regions with hypermutations are highlighted with upward black arrows and can also be observed by looking for a straight line made of SNV depicting colored dots, each panel showing mutation of each sample (A-D).

**Supplementary Figure-3: Comparison of our patient data with TCGA cohorts of different kinds of cancer.** Mutation load from TCGA MC3 was taken advantage of to contrast mutation burden in our study with 33 TCGA cohorts. Each curved line depicts all the samples of one cohort. The red line in each cohort plot means the average mutation burden of patients of that cancer. All of our samples (4) were included in this cohort comparison; our study being named “DU_BLCA”

Supplemetary Table 1: Per sample BAM metrics

| Sample | Total Reads | Mapped % | Properly Paired % | Duplication rate% | On-Target Rate | >=20× | >=30× | MEDIAN INSERT SIZE | MEAN INSERT SIZE |
| --- | --- | --- | --- | --- | --- | --- | --- | --- | --- |
| DU_001 | 43565893 | 99.95 | 98.28 | 6.62 | 0.637 | 0.751 | 0.577 | 219 | 236.243 |
| DU_002 | 39967236 | 99.97 | 98.57 | 6.75 | 0.453 | 0.712 | 0.525 | 213 | 230.548 |
| DU_003 | 27502339 | 99.93 | 99.15 | 6.88 | 0.619 | 0.672 | 0.495 | 200 | 213.770 |
| DU_004 | 42959142 | 99.95 | 98.4 | 6.95 | 0.640 | 0.749 | 0.572 | 220 | 238.343 |
| DU_T_001 | 37415252 | 99.95 | 98.5 | 6.79 | 0.627 | 0.684 | 0.506 | 214 | 230.324 |
| DU_T_002 | 36919860 | 99.95 | 98.35 | 6.83 | 0.631 | 0.677 | 0.493 | 216 | 232.975 |
| DU_T_003 | 22747569 | 99.97 | 98.44 | 6.58 | 0.625 | 0.722 | 0.553 | 212 | 226.841 |
| DU_T_004 | 42959142 | 99.95 | 98.4 | 7.02 | 0.616 | 0.675 | 0.504 | 221 | 238.987 |

Supplimentary Table 2: Per sample tumor purity estimates by Sequenza

| **Sample** | **Purity (Cellularity)** | **Ploidy** | **SLPP Score (Highest shown)** |
| --- | --- | --- | --- |
| DU_T_001.final | 0.11 (11%) | 2.3 | 0.03359 |
| DU_T_002.final | 0.22 (22%) | 5.8 | 0.01829 |
| DU_T_003.final | 0.10 (10%) | 2.5 | 0.01980 |
| DU_T_004.final | 0.28 (28%) | 3.9 | 0.02060 |

Supplementary Table 3: Comparison of mutation with TCGA

| **cBioPortal** | | | | | **BDUC samples** | | |
| --- | --- | --- | --- | --- | --- | --- | --- |
| Gene | Total Mutation count | Sample count | Profiled Samples | Frequency | ns SNV count | Sample count | Frequency |
| TP53 | 881 | 741 | 1582 | 46.80% | 3 | 1 | 25% |
| TTN | 881 | 482 | 1237 | 39.00% | 3 | 3 | 75% |
| KDM6A | 444 | 406 | 1485 | 27.30% | 1 | 1 | 25% |
| MUC16 | 450 | 299 | 1138 | 26.30% | 9 | 3 | 75% |
| KMT2D | 548 | 389 | 1485 | 26.20% | 1 | 1 | 25% |
| ARID1A | 462 | 364 | 1485 | 24.50% | 0 | 0 | 0 |
| ZNF729 | 17 | 17 | 72 | 23.60% | 0 | 0 | 0 |
| PIK3CA | 365 | 338 | 1582 | 21.40% | 0 | 0 | 0 |
| ANKRD62 | 15 | 13 | 72 | 18.10% | 0 | 0 | 0 |
| RB1 | 312 | 265 | 1582 | 16.80% | 0 | 0 | 0 |
| RYR2 | 205 | 186 | 1138 | 16.30% | 3 | 3 | 75% |
| SYNE1 | 252 | 201 | 1237 | 16.20% | 0 | 0 | 0 |
| TERT | 212 | 202 | 1264 | 16.00% | 0 | 0 | 0 |
| KMT2C | 325 | 234 | 1485 | 15.80% | 4 | 3 | 75% |
| FGFR3 | 276 | 241 | 1582 | 15.20% | 1 | 1 | 25% |
| EP300 | 271 | 219 | 1485 | 14.70% | 0 | 0 | 0 |
| HMCN1 | 189 | 172 | 1237 | 13.90% | 0 | 0 | 0 |
| IRX3 | 10 | 10 | 72 | 13.90% | 0 | 0 | 0 |
| DNAH10OS | 11 | 10 | 72 | 13.90% | 0 | 0 | 0 |
| FLG | 193 | 166 | 1237 | 13.40% | 11 | 4 | 100% |
| CREBBP | 211 | 189 | 1485 | 12.70% | 1 | 1 | 25% |
| STAG2 | 207 | 189 | 1485 | 12.70% | 0 | 0 | 0 |
| ERBB2 | 223 | 188 | 1485 | 12.70% | 2 | 1 | 25% |
| CDK2AP1 | 9 | 9 | 72 | 12.50% | 0 | 0 | 0 |
| ERBB3 | 267 | 178 | 1485 | 12.00% | 1 | 1 | 25% |
| SYNE2 | 186 | 146 | 1237 | 11.80% | 2 | 2 | 50% |
| FAT4 | 193 | 152 | 1288 | 11.80% | 2 | 2 | 50% |
| FAT1 | 198 | 164 | 1386 | 11.80% | 0 | 0 | 0 |
| ATM | 211 | 169 | 1485 | 11.40% | 3 | 1 | 25% |
| NEB | 139 | 127 | 1138 | 11.20% | 0 | 0 | 0 |
| OBSCN | 144 | 126 | 1138 | 11.10% | 2 | 1 | 25% |
| BIRC6 | 188 | 137 | 1237 | 11.10% | 1 | 1 | 25% |
| WDR87 | 11 | 8 | 72 | 11.10% | 1 | 1 | 25% |
| KMT2A | 203 | 165 | 1485 | 11.10% | 1 | 1 | 25% |
| CSMD3 | 149 | 122 | 1107 | 11.00% | 1 | 1 | 25% |
| AHNAK2 | 134 | 123 | 1138 | 10.80% | 19 | 4 | 100% |
| DNAH11 | 130 | 122 | 1138 | 10.70% | 1 | 1 | 25% |
| MUC17 | 180 | 131 | 1237 | 10.60% | 14 | 2 | 50% |
| MACF1 | 168 | 131 | 1237 | 10.60% | 3 | 2 | 50% |
| ZFHX4 | 134 | 120 | 1138 | 10.50% | 1 | 1 | 25% |
| XIRP2 | 144 | 119 | 1165 | 10.20% | 1 | 1 | 25% |
| SPTAN1 | 190 | 126 | 1237 | 10.20% | 1 | 1 | 25% |
| ERCC2 | 154 | 150 | 1485 | 10.10% | 2 | 2 | 50% |
